# Optimal donor for severe aplastic anemia patient requiring allogeneic hematopoietic stem cell transplantation: A large-sample study from China

**DOI:** 10.1038/s41598-018-20853-9

**Published:** 2018-02-06

**Authors:** Yunjing Zeng, Sanbin Wang, Jishi Wang, Li Liu, Yi Su, Zhixiang Lu, Xuemei Zhang, Yanqi Zhang, Jiang Fan Zhong, Lihui Peng, Qiang Liu, Yinghao Lu, Lei Gao, Xi Zhang

**Affiliations:** 1 Department of Hematology, Xinqiao Hospital, Third Military Medical University, Chongqing, China; 2 Department of Hematology, General Hospital of Kunming Military Region of PLA, Kunming, China; 3 Department of Hematology, Affiliated Hospital of Guizhou Medical University, Guiyang, China; 4 Department of Hematology, Tangdu Hospital, Forth Military Medical University, Xi’an, China; 5 Department of Hematology, General Hospital of Chengdu Military Region of PLA, Chengdu, Sichuan China; 6 Department of Hematology, First Yunnan Provincial People’s Hospital, Kunming, China; 7 Department of Hematology, Affiliated Hospital of Kunming Medical College, Kunming, China; 8 Department of Health Statistics, College of Military Preventive Medicine, Third Military Medical University, Chongqing, China

## Abstract

HLA-haploidentical hematopoietic stem cell transplantation (HSCT) may be an option for severe aplastic anemia (SAA) patients. However, to date, no large-sample studies have been performed to determine which types of SAA patients are suitable for HLA-haploidentical HSCT. We retrospectively studied 189 consecutive patients with SAA who underwent HLA-identical or HLA-haploidentical HSCT at seven transplant centers in China. Propensity score matching (PSM) was applied in this study to reduce the influence of potential confounders. The 5-year overall survival (OS) rate was 72.0% in the HLA-haploidentical group and 76.5% in the HLA-identical group. The median time to achieve engraftment and the incidence of acute GVHD/chronic GVHD were not significantly different between the two groups. In the subgroup analysis, the outcome of patients older than 40 years in the HLA-haploidentical group was significantly poorer than that of patients younger than 40 years in the same group and that of patients older than 40 years in the HLA-identical group. Based on the above results, we suggest that HLA-haploidentical relative HSCT should be considered as a valid alternative option for patients younger than 40 years with SAA for whom no matched sibling donor is available.

## Introduction

Aplastic anemia (AA) is a life-threatening bone marrow failure syndrome that is defined as pancytopenia with hypocellular bone marrow in the absence of an abnormal infiltrate or fibrosis. A severe aplastic anemia (SAA) diagnosis is given when patients meet at least 2 of the following criteria: an absolute neutrophil count (ANC) of less than 0.5 × 10^9^/L, a platelet count (PLT) of less than 20 × 10^9^/L, or a corrected reticulocyte count (CRC) of less than 1%^1^. Standard treatments for SAA include immunosuppressive therapy (IST) and human leukocyte antigen (HLA)-identical sibling hematopoietic stem cell transplantation (HSCT)^1–5^. A study from Europe reported the outcomes of 563 children with SAA and showed that overall survival (OS) after upfront HSCT from an HLA-matched family donor was comparable to that after IST but event-free survival (EFS) was superior after upfront HSCT^6^. Another study from Japan reported similar results^7^. Furthermore, a report from the European Society for Blood and Marrow Transplantation (EBMT) indicated that the actuarial 10-year survival following first-line bone marrow transplantation (BMT) was superior to that post-IST^8^. The 2009 and 2015 British AA guidelines recommended HLA-identical sibling HSCT as the initial treatment of choice for newly diagnosed patients <40 years old. Unfortunately, the chance of finding an HLA-identical sibling donor is only 25%, and unrelated donors cannot be identified in time for all patients who need an allograft^9^. Patients who lack a matched sibling or unrelated donor require other graft sources. HLA-haploidentical relative HSCT has been greatly improved during the past decade^10,11^. Many cases of mismatched related donor transplants in SAA patients have been reported^9,12–21^. The latest 2015 British AA guidelines recommended alternative donor HSCT as an experimental treatment for patients who lacked suitably matched donors^1^. However, few reports have compared the prognosis between HLA-identical sibling and HLA-haploidentical relative HSCT^22^.

To evaluate the efficacy and safety of HLA-haploidentical relative HSCT for SAA, a long-term retrospective cohort clinical study was conducted at seven transplant centers in western China. This trial investigated and compared the long-term survival, hematopoietic reconstitution time, incidence of graft versus host disease (GVHD) and infection, and graft failure rate between HLA-identical sibling and HLA-haploidentical relative HSCT.

## Results

### Baseline and transplant-related characteristics

As shown in Table 1, when comparing the entire cohort, we found that patients were younger and the donors were older in the HLA-haploidentical HSCT group than those in the HLA-identical HSCT group. All the patients received IST before HSCT: 5.4% (11/115) in the HLA-haploidentical group and 9.6% (4/74) in the HLA-identical group were treated with antithymocyte globulin (ATG) + cyclosporine A (CsA), but most patients received only CsA and/or corticosteroids. The conditioning regimen and GVHD prophylaxis options were different; more patients accepted the BU + CY conditioning regimen in the HLA-haploidentical group, whereas the patients in the HLA-identical group accepted only the CY or Flu + CY conditioning regimen.

**Table 1:** Comparison of patient characteristics between the HLA-haploidentical and HLA-identical hematopoietic stem cell transplantation groups.

Characteristics	Before matching				After matching			
	HLA-haploidentical HSCT group	HLA-identical HSCT group	Statistics	*P* value	HLA-haploidentical HSCT group	HLA-identical HSCT group	Statistics	*P* value
Number of patients	115	74			70	70		
Patient age, median (range)	18 (3–58)	27.5 (3–53)	−4.082*	0.000	21.0 (5.0–58.0)	26.5 (33.0–53.0)	−1.760*	0.078
Patient age composition			17.070^†^	0.000			3.197^†^	0.202
<20	68 (59.1%)	22 (29.7%)			32 (45.7%)	22 (31.4%)		
20–40	39 (33.9%)	38 (51.4%)			30 (42.9%)	36 (51.4%)		
≥40	8 (7.0%)	14 (18.9%)			8 (11.4%)	12 (17.1%)		
Patient gender, male/female	77 (67.0%)/38 (33.0%)	43 (58.1%)/31 (41.9%)	1.521*	0.217	42 (60.0%)/28 (40.0%)	41 (58.6%)/29 (41.4%)	0.030*	0.863
Interval from diagnosis to HSCT (month), median (range)	3.0 (1.0–168.0)	3.0 (1.0–181.0)	−0.352*	0.724	3.0 (1.0–168.0)	3.0 (1.0–181.0)	−0.514*	0.607
Donor age, median (range)	38.0 (11.0–64.0)	30.0 (3.0–52.0)	−3.194*	0.001	37.0 (11.0–64.0)	28.0 (3.0–52.0)	−1.996*	0.046
Donor gender, male/female	73 (63.5%)/42 (36.5%)	40 (51.5%)/34 (45.9%)	1.663^†^	0.197	41 (58.6%)/29 (41.4%)	38 (54.3%)/32 (45.7%)	0.261^†^	0.609
ABO blood type			3.471^†^	0.482			3.487^†^	0.480
Match	61 (53.0%)	46 (62.2%)			37 (52.9%)	43 (61.4%)		
Minor mismatch	25 (21.7%)	10 (13.5%)			15 (21.4%)	10 (14.3%)		
Major mismatch	24 (20.9%)	13 (17.6%)			14 (20.0%)	13 (18.6%)		
Major and minor mismatch	2 (1.7%)	3 (4.1%)			1 (1.4%)	3 (4.3%)		
Unknown	3 (2.6%)	2 (2.7%)			3 (4.3%)	1 (1.4%)		
**Infused cell dose**								
PBSC (×10^8^/kg), median (range)	8.85 (2.30–26.00)	8.14 (0.84–26.10)	−1.087*	0.277	7.60 (2.42–25.10)	8.14 (0.84–26.10)	−0.256*	0.798
BMNC (×10^8^/kg), median (range)	3.55 (0.00–17.5)	3.50 (0.41–13.00)	−0.865*	0.387	3.50 (0.35–17.50)	3.40 (0.41–13.00)	−0.785*	0.432
CD34+ cells (×10^6^/kg), median (range)	7.21 (1.61–24.50)	6.27 (1.02–35.00)	−1.442*	0.149	6.75 (1.61–19.90)	6.27 (2.02–35.00)	−1.110*	0.267
Conditioning regimen			15.669^†^	0.000			5.067^†^	0.079
Flu + CY	74 (64.3%)	54 (73.0%)			48 (68.6%)	5 (74.3%)		
Bu + CY	36 (31.3%)	8 (10.8%)			17 (24.3%)	8 (11.4%)		
CY	5 (4.3%)	12 (16.2%)			5 (7.1%)	10 (14.3%)		
GVHD prophylaxis			6.182^†^	0.045			1.299^†^	0.522
CsA + MMF + MTX	80 (69.6%)	62 (83.8%)			55 (78.6%)	58 (82.9%)		
FK506 + MMF + MTX	22 (19.1%)	5 (6.8%)			9 (12.9%)	5 (7.1%)		
PT-CY	13 (11.3%)	7 (9.5%)			6 (8.6%)	7 (10.0%)		
**Previous transfusions**								
RBC (U), median(range)	6.0 (0.0–100.0)	6.0 (0.0–56.0)	0.042*	0.966	6.0 (0.0–92.0)	6.0 (0.0–56.0)	0.004*	0.997
PLT (U), median(range)	8.0 (0.0–180.0)	8.0 (0.0–150)	1.082*	0.279	8.0 (0.0–180.0)	8.0 (0.0–150)	0.773*	0.440
**CBCs before HSCT**								
ANC (×10^9^/L), median(range)	0.2 (0.0–0.6)	0.3 (0.0–0.9)	1.342*	0.180	0.2 (0.0–0.6)	0.3 (0.0–0.9)	1.545*	0.122
HGB (g/L), median(range)	66.0 (43.0–95.0)	66.0 (40.0–90.0)	0.553*	0.580	69.0 (43.0–95.0)	66.0 (40.0–90.0)	1.270*	0.204
PLT (×10^9^/L), median(range)	8.0 (0.0–20.0)	10.0 (0.0–20.0)	0.647*	0.518	7.0 (0.0–20.0)	10.0 (0.0–20.0)	0.831*	0.406
RET (×10^9^/L), median(range)	9.0 (0.0–21.0)	11.0 (0.0–20.0)	1.247*	0.212	8.0 (0.0–21.0)	11.0 (0.0–20.0)	1.780*	0.075
Previous IST			1.066^†^	0.302			0.888^†^	0.346
ATG, n (%)	11 (9.6)	4 (5.4)			7 (10.0)	4 (5.7)		
Glucocorticoids and/or CsA, n (%)	104 (90.4)	70 (94.6)			63 (90.0)	66 (94.3)		

PBSC, peripheral blood stem cell; BMSC, bone marrow stem cell; PT-CY, post-transplant cyclophosphamide; RBC, red blood cells; PLT, platelets; HGB, hemoglobin; CBCs: complete blood counts; ANC: absolute neutrophil count; RET: reticulocyte count; ATG: antithymocyte globulin; and CsA: cyclosporine A

*z value from the Mann-Whitney test. ^†^Chi-square from the Chi-squared test.

Propensity score matching (PSM) analysis created 70 pairs of patients. Comparisons of the patient characteristics between the HLA-haploidentical and HLA-identical groups in the propensity-matched cohort are shown in Table 1. All the variables were balanced between the two groups (p > 0.05).

### Survival

**Figure 1:**
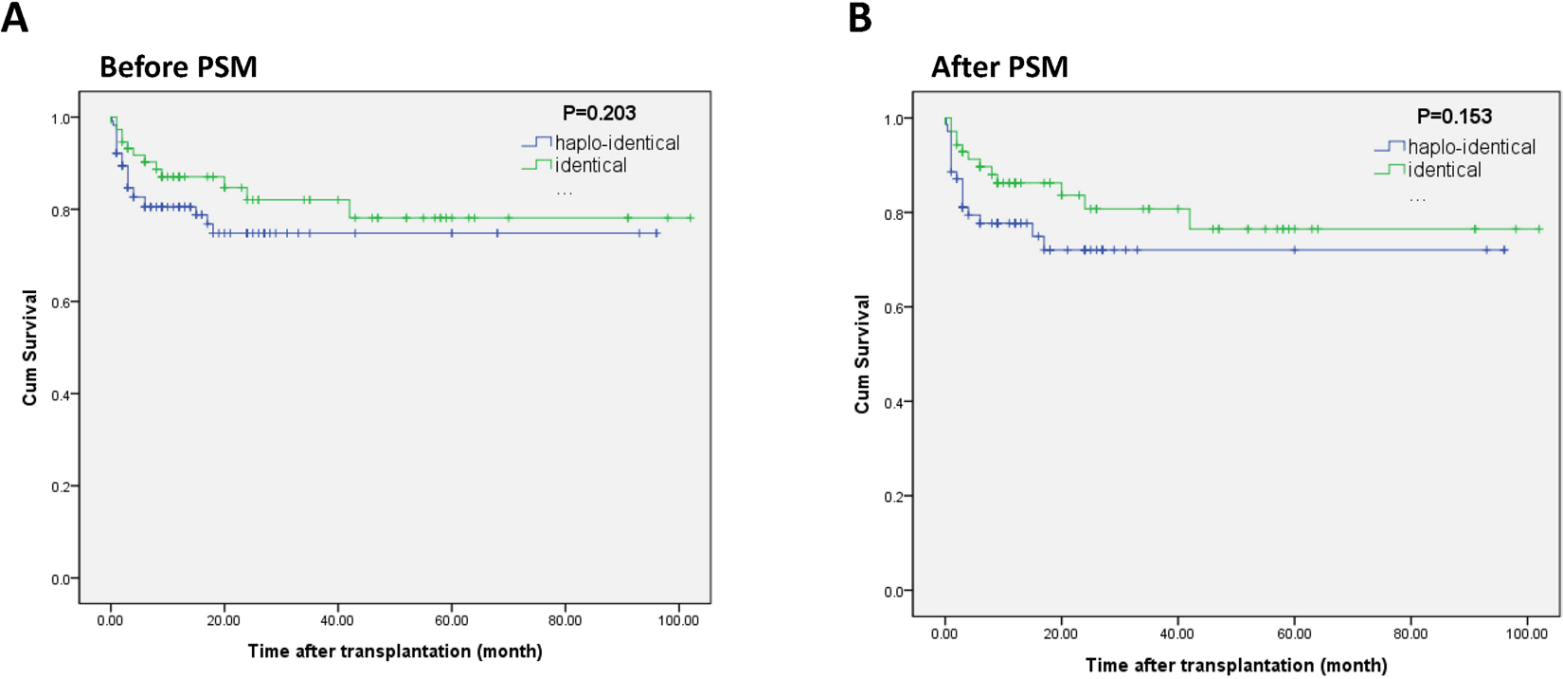
Overall survival of severe aplastic anemia patients after hematopoietic stem cell transplantation. (**A**) The OS of the HLA-haploidentical group and HLA-identical group before propensity score matching. (**B**) The OS of the HLA-haploidentical group and HLA-identical group after propensity score matching.

The 5-year OS rate was 74.8% in the HLA-haploidentical group versus 78.1% in the HLA-identical group before PSM. No significant difference was observed between the groups (χ^2^ = 1.624, *p* = 0.203) (Fig. 1A). After PSM, the overall survival of the two groups was still comparable; the estimated 5-year OS rate was 72.0% in the HLA-haploidentical group and 76.5% in the HLA-identical group (χ^2^ = 2.044, *p* = 0.153) (Fig. 1B).

**Figure 2:**
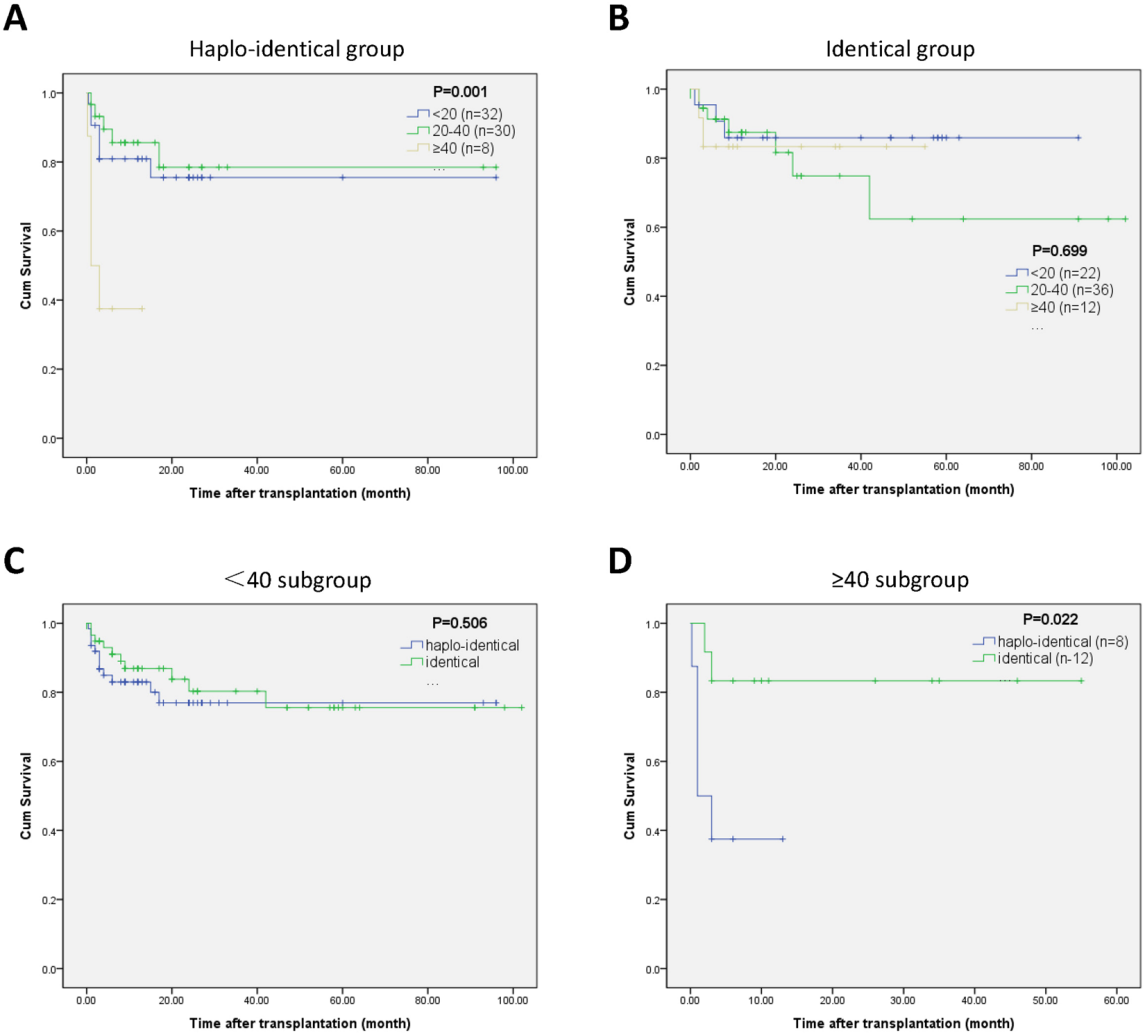
Overall survival of severe aplastic anemia patients after hematopoietic stem cell transplantation by age. (**A**) Subgroup analysis by age in the HLA-haploidentical group. (**B**) Subgroup analysis by age in the HLA-identical group. (**C**) Overall survival of patients under 40 years of age in the HLA-haploidentical and HLA-identical groups. (**D**) Overall survival of patients 40 years of age and above in the HLA-haploidentical and HLA-identical groups.

In the subgroup analysis, we classified all the propensity score-matched patients into three age levels (<20 years, 20–40 years, and ≥40 years). In the HLA-haploidentical group, the OS rates of the three age levels were different (χ^2^ = 13.020, *p* = 0.001) (Fig. 2A). The estimated 5-year OS rate was 75.5% among patients <20 years old (n = 32), and the estimated 1-year OS rates among patients aged 20–40 years old (n = 30) and patients aged ≥40 years old (n = 8) were 78.5% and 37.5%, respectively. However, in the HLA-identical group, the OS rates of the three age levels were similar (χ2 = 0.717, *p* = 0.699) (Fig. 2B). The estimated 1-year OS rates of patients <20 years old and patients 20–40 years old were 85.9% and 62.4%, respectively, and the estimated 4-year OS rate was 83.3% among patients ≥40 years old (n = 12). Next, we compared the two transplant groups at the same age level. At the <40-years level, the 8-year OS rates were 76.9% and 75.6% in the HLA-haploidentical and HLA-identical groups, respectively (χ^2^ = 0.443, *p* = 0.506) (Fig. 2C). However, in the ≥40-years level, the outcome of the HLA-haploidentical group was significantly poorer than the outcome of the HLA-identical group (χ^2^ = 5.210, *p* = 0.022) (Fig. 2D). The estimated 1-year OS rate was 37.5% in the HLA-haploidentical group, whereas the 4-year OS was 83.3% in the HLA-identical group.

**Figure 3:**
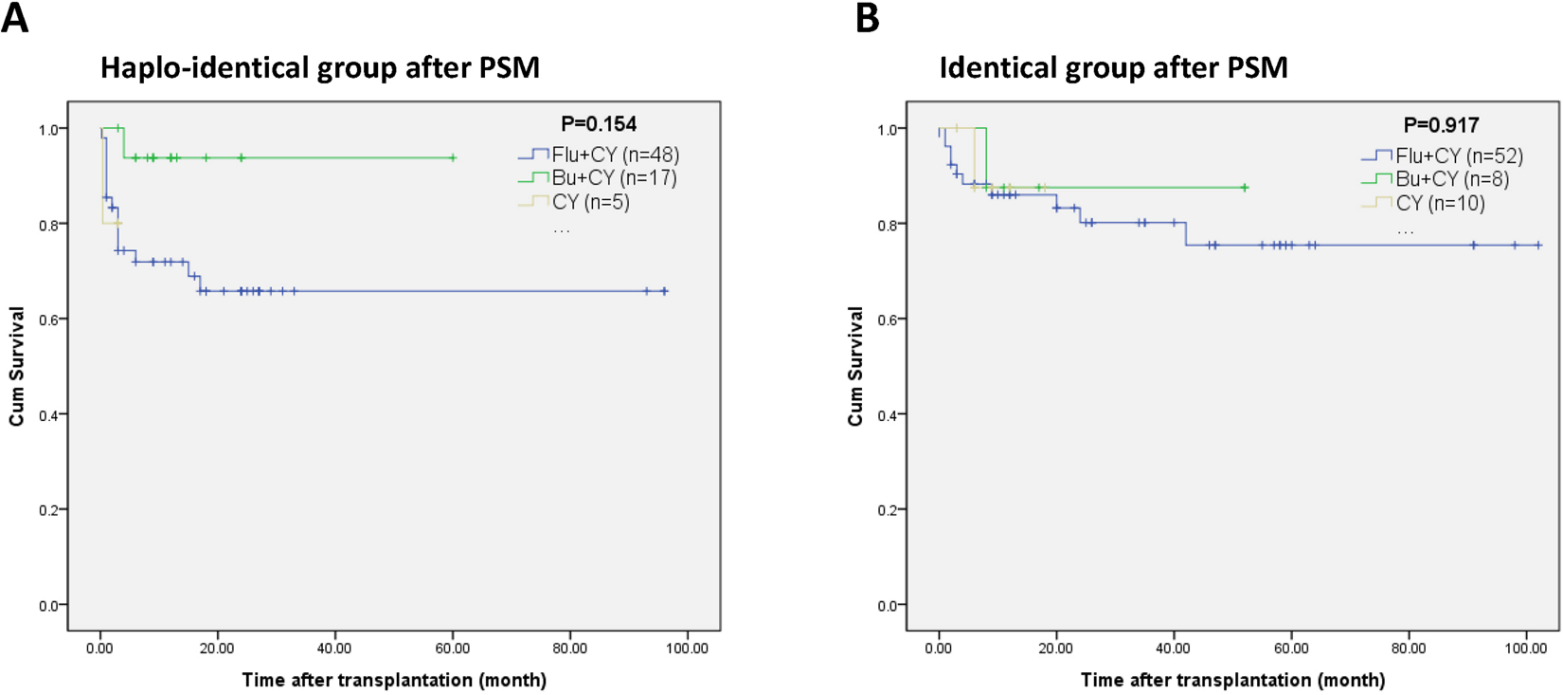
Overall survival of severe aplastic anemia patients after hematopoietic stem cell transplantation by conditioning regimen. (**A**) Subgroup analysis by conditioning regimen in the HLA-haploidentical group. (**B**) Subgroup analysis by conditioning regimen in the HLA-identical group.

To analyze the effects of the conditioning regimen on OS, we divided the patients into three subgroups according to the conditioning regimen protocol (Flu + CY, BU + CY, and CY). In both the HLA-haploidentical and HLA-identical HSCT groups, the OS rates were similar among the three conditioning regimen subgroups. In the HLA-haploidentical group, the 8-year OS rate in the Flu + CY subgroup was 68.5%, the 5 year OS rate in the BU + CY subgroup was 93.8%, and the 1-year OS rate in the CY subgroup was 80.8% (χ^2^ = 3.739, *p* = 0.154) (Fig. 3A). In the HLA-identical group, the 8-year OS rate in the Flu + CY subgroup was 75.4%, the 5 year OS rate in the BU + CY subgroup was 87.5%, and the 2-year OS rate in the CY subgroup was 87.5% (χ^2^ = 0.174, *p* = 0.917) (Fig. 3B).

### Hematopoietic reconstitution

In the HLA-haploidentical group, 103/115 (89.6%) patients achieved hematopoietic reconstitution versus 71/74 (95.9%) patients in the HLA-identical group (χ^2^ = 2.509, *p* = 0.113). The median time intervals to achieve neutrophil engraftment and platelet engraftment were 13.0 days versus 13.0 days (*p* = 0.200) and 14.0 days versus 14.0 days (*p* = 0.351), respectively (Table 2). After PSM, 61/70 patients and 68/70 patients achieved hematopoietic reconstitution in the HLA-haploidentical and HLA-identical groups, respectively (Fisher’s exact test, *p* = 0.055). The median times to achieve neutrophil and platelet engraftment were not significantly different between the two groups (Table 2).

**Table 2:** Time to engraftment.

Characteristics	Before matching				After matching			
	HLA-haploidentical HSCT group	HLA-identical HSCT group	Statistics	*P* value	HLA-haploidentical HSCT group	HLA-identical HSCT group	Statistics	*P* value
NEU (day), median (range)	13.0 (9.0–25.0)	13.0 (7.0–25.0)	−1.283	0.200	13.0 (9.0–25.0)	13.0 (7.0–25.0)	−1.291	0.197
PLT (day), median (range)	14.0 (8.0–82.0)	14.0 (9.0–34.0)	−0.932	0.351	14.0 (8.0–82.0)	14.0 (9.0–34.0)	−0.511	0.609

NEU: neutrophils; and PLT: platelets.

### GVHD

**Figure 4:**
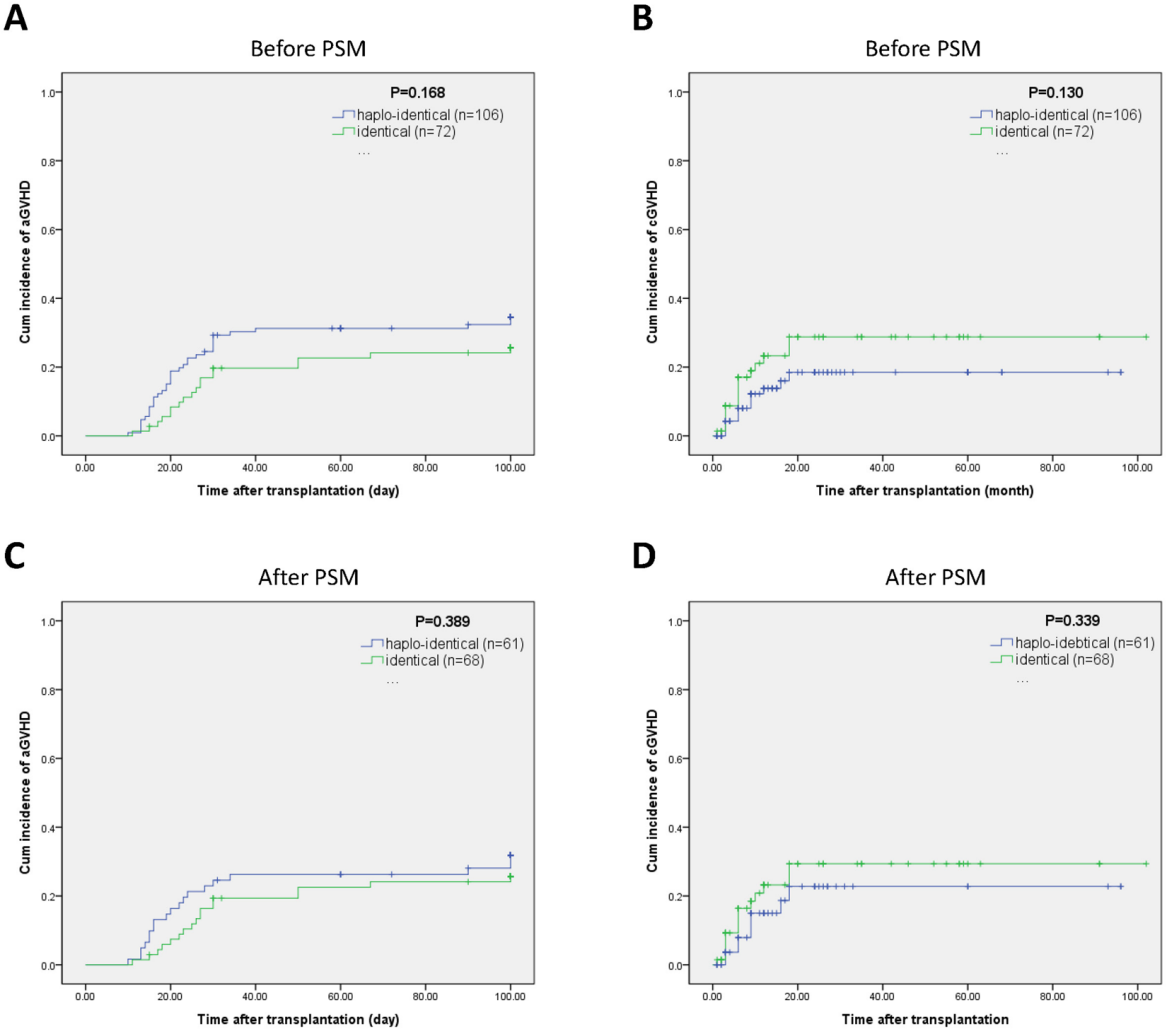
Graft versus host disease incidence among severe aplastic anemia patients after hematopoietic stem cell transplantation. (**A**) The cumulative incidence rates of acute graft versus host disease in the HLA-haploidentical and HLA-identical groups before propensity score matching. (**B**) The cumulative incidence rates of chronic graft versus host disease in the HLA-haploidentical and HLA-identical groups before propensity score matching. (**C**) The acute graft versus host disease rates in the HLA-haploidentical and HLA-identical groups after propensity score matching. (**D**) The cumulative incidence rates of chronic graft versus host disease in the HLA-haploidentical and HLA-identical groups after propensity score matching.

The cumulative incidence rates of all aGVHD cases at 100 days were 34.5% and 25.7% in the HLA-haploidentical and HLA-identical groups, respectively (χ^2^ = 1.898, *p* = 0.168) (Fig. 4A). The cumulative incidence rates of 5-year cGVHD in the HLA-haploidentical and HLA-identical groups were 18.5% and 28.8%, respectively (χ^2^ = 2.297, *p* = 0.130) (Fig. 4B). After PSM, the aGVHD rates at 100 days in the HLA-haploidentical and HLA-identical groups were 31.9% and 25.7%, respectively (χ2 = 0.741, *p* = 0.389) (Fig. 4C), and the cumulative incidence rates of 5 year cGVHD were 22.8% and 23.2%, respectively (χ^2^ = 0.913, *p* = 0.339) (Fig. 4D).

**Figure 5:**
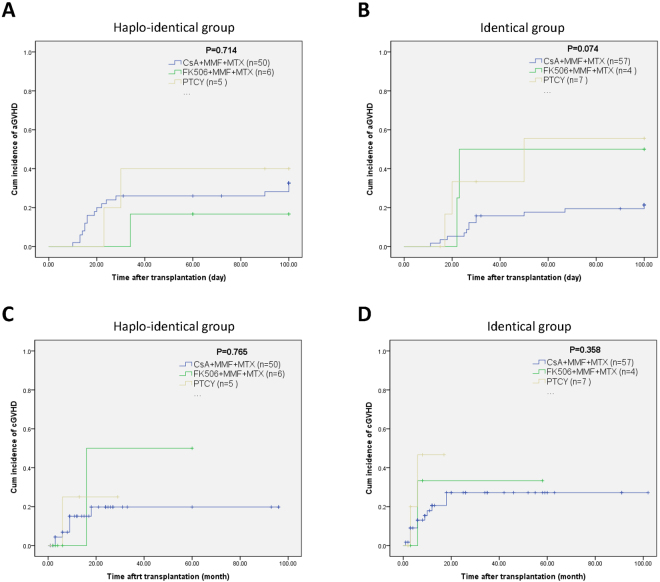
Graft versus host disease incidence among severe aplastic anemia patients after hematopoietic stem cell transplantation by prophylaxis regimen. (**A**) Subgroup analysis of acute graft versus host disease by prophylaxis regimen in the HLA-haploidentical group. (**B**) Subgroup analysis of acute graft versus host disease by prophylaxis regimen in the HLA-identical group. (**C**) Subgroup analysis of chronic graft versus host disease by prophylaxis regimen in the HLA-haploidentical group. (**D**) Subgroup analysis of chronic graft versus host disease by prophylaxis regimen in the HLA-identical group.

Next, we divided the propensity score-matched patients into 3 subgroups according to their GVHD prophylaxis regimen (CsA + MMF + MTX, FK506 + MMF + MTX and PT-CY). The cumulative incidence rates of aGVHD at + 100 days were 32.5%, 16.7% and 40.0% in the CsA + MMF + MTX, FK506 + MMF + MTX and PT-CY HLA-haploidentical HSCT subgroups, respectively (χ^2^ = 0.673, *p* = 0.714) (Fig. 5A), and 21.3%, 50.0% and 55.6% in the corresponding HLA-identical HSCT subgroups, respectively (χ^2^ = 5.205, *p* = 0.074) (Fig. 5B). The cumulative incidence rates of cGVHD were 19.8% at 8 years, 50.0% at 5 years, and 25.0% at 2 years for each HLA-haploidentical HSCT subgroup (χ^2^ = 0.536, *p* = 0.765) (Fig. 5C) and 27.2% at 8 years, 33.3% at 5 years, and 46.7% at 2 years for each HLA-identical HSCT subgroup (χ^2^ = 2.055, *p* = 0.358) (Fig. 5D).

### Infection

**Figure 6:**
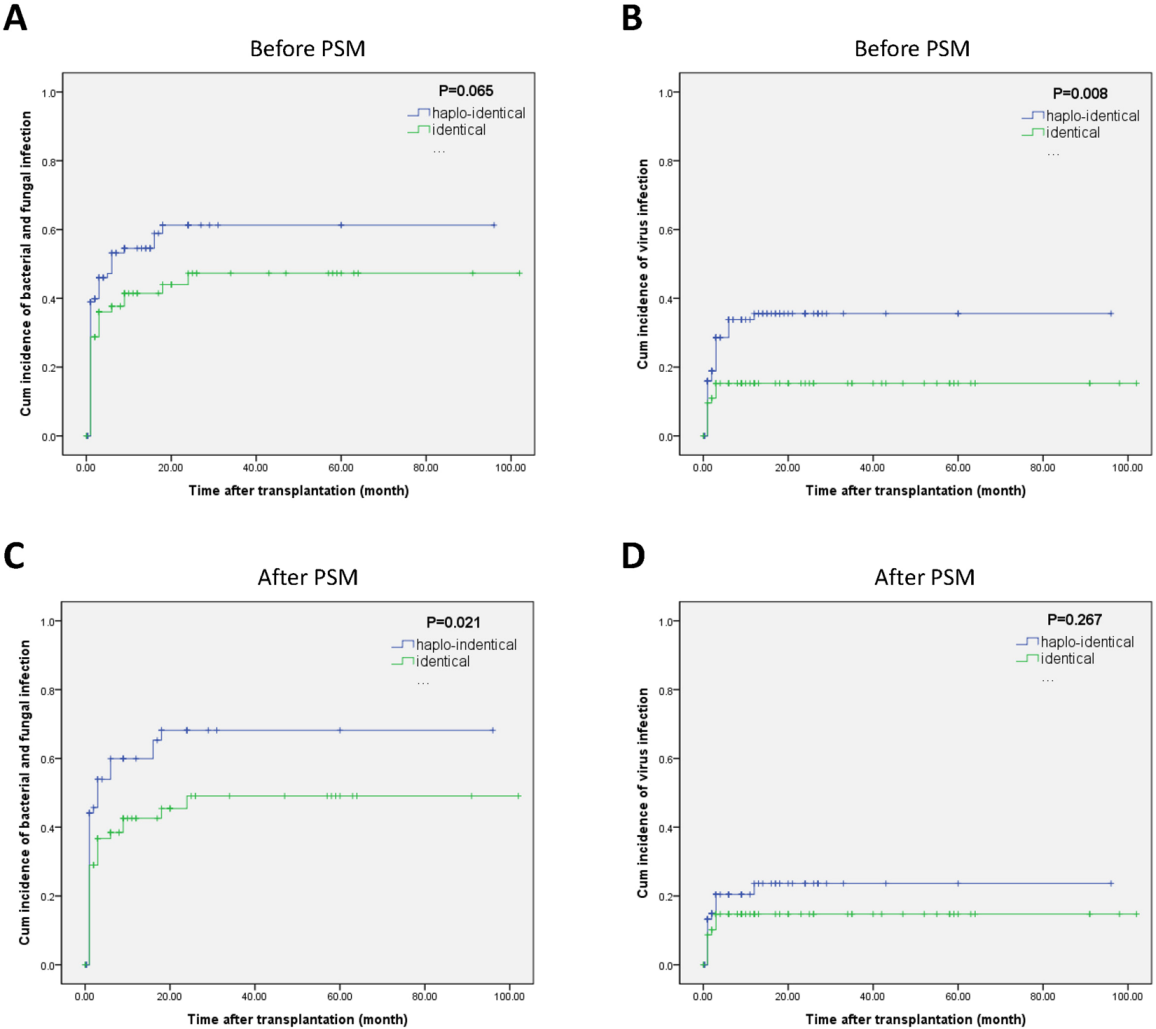
Infection rates among severe aplastic anemia patients after hematopoietic stem cell transplantation. (**A**) The cumulative incidence of bacterial and fungal infections before propensity score matching. (**B**) The cumulative incidence of CMV infection before propensity score matching. (**C**) The cumulative incidence of bacterial and fungal infections after propensity score matching. (**D**) The cumulative incidence of CMV infection after propensity score matching.

The cumulative incidence rates of bacterial and fungal infections at 5 years were 61.3% in the HLA-haploidentical group and 47.2% in the HLA-identical group (χ^2^ = 3.414, *p* = 0.065) (Figure A). The cumulative incidence of cytomegalovirus (CMV) infection at 1 year in the HLA-haploidentical group was significantly higher than that in the HLA-identical group (35.6% versus 15.3%; χ^2^ = 7.052, *p* = 0.008) (Fig. 6B). After PSM, the cumulative incidence rates of bacterial and fungal infections at 5 years in the HLA-haploidentical group were significantly higher than those in the HLA-identical group (68.2% versus 36.7%, χ^2^ = 5.365, *p* = 0.021) (Fig. 6C). The cumulative incidence rates of CMV infection at 1 year in the HLA-haploidentical and HLA-identical groups were 23.6% and 14.7%, respectively (χ^2^ = 1.232, *p* = 0.267) (Fig. 6D).

### Multivariate Cox regression

In the multivariate regression analysis, survival outcome was significantly adversely associated with red blood cell (RBC) transfusions before HSCT (p = 0.001, HR = 0.026, 95% CI 1.010–1.042) and patient age (p = 0.049, HR = 1.708, 95% CI 1.002–2.914). We did not observe differences in survival outcomes based on donor type (Table 3).

**Table 3:** Multivariate Cox regression analysis of risk factors affecting survival among hematopoietic stem cell transplantation patients with severe aplastic anemia (using forward stepwise regression)

Characteristics	Before matching			After matching		
	*P* value	HR	95.0% CI for HR	*P* value	HR	95.0% CI for HR
HLA-identical group	0.372	0.724	0.357–1.471	0.202	0.611	0.287–1.302
GVHD prophylaxis	0.027	1.601	1.056–2.426	0.014	1.811	1.130–2.904
Patient age	0.036	1.690	1.036–2.755	0.049	1.708	1.002–2.914
Previous RBC transfusion (U)	0.026	1.016	1.002–1.031	0.004	1.024	1.008–1.041

GVHD: graft versus host disease; and RBC: red blood cells.

## Discussion

The aim of this study was to compare the outcomes of HLA-haploidentical relative HSCT and HLA-identical sibling HSCT for SAA patients. To the best of our knowledge, this study enrolled the largest number of patients for the comparison of outcomes between HLA-haploidentical relative HSCT and HLA-identical sibling HSCT using similar doses and graft compositions and identical conditioning regimens. The results suggested that, with the exception of a higher incidence of bacterial and fungal infections, HLA-haploidentical relative HSCT was comparable to HLA-identical sibling HSCT in terms of OS, incidence rates of aGVHD and cGVHD, and time to engraftment.

In our retrospective study, we found that the OS rates of the HLA-haploidentical and HLA-identical HSCT groups were all greater than 70% after PSM. These results were different from those of Gupta’s study in 2010^23^ and were indicative of the rapid advances in transplantation technology^24,25^. In the HLA-identical sibling HSCT group, the SAA patients in different age groups exhibited similar survival rates. Thus, in the 2015 edition of the British Guide for SAA^1^, the age limit for hematopoietic stem cell transplantation in SAA patients was expanded to 50 years, and the 40-year-old boundary became increasingly blurred for HLA-identical HSCT^26,27^. HLA-haploidentical relative HSCT was the first alternative transplantation regimen to be considered among other treatment options after failure to respond to IST in the absence of an HLA-identical sibling donor or a suitably matched unrelated donor^1^. Under those circumstances, who are the suitable donors for HLA-haploidentical HSCT for the treatment of SAA? In our study, the prognosis was significantly better among patients younger than 40 years and was equal to the prognosis achieved with HLA-identical sibling HSCT. In contrast, patients older than 40 years had a significantly poorer prognosis, with 5/8 patients older than 40 years in the HLA-haploidentical group dying in the first 6 months after transplantation. The direct causes of death were infection (4/5) and heart failure (1/5), and 4 of these patients had first graft failure. The relatively high rates of graft failure- and transplant-related death were quite different from the rates in elderly patients with hematological malignancies who underwent HLA-haploidentical HSCT^28^. Thus, the 40-year-old boundary remains of great significance in HLA-haploidentical HSCT. Moreover, HLA-haploidentical relative HSCT should be considered as an alternative transplantation option for SAA patients younger than 40 years.

The hematopoietic engraftment rate and time to engraftment of the HLA-haploidentical and HLA-identical groups were comparable to that in previous reports^5,9,29–31^. The cumulative incidence of aGVHD in the HLA-haploidentical group was slightly higher than that in the HLA-identical group, whereas the cumulative incidence of cGVHD was lower in the HLA-haploidentical group than in the HLA-identical group; however, the differences were not statistically significant. Notably, both groups had higher incidence rates of aGVHD and lower incidence rates of cGVHD compared with the rates observed in our previous report^15^ and other recently published studies^6,26^; these differences may be attributed to the different diagnostic criteria or the enrollment of older patients in our study. Among the three different GVHD prophylaxis subgroups, we observed no significant differences in the GVHD incidence rates. There was a lower incidence of grade II to IV aGVHD in the PT-CY subgroup, but the sample size was too small; a study with a larger sample size is required to draw a reliable conclusion. The incidence of bacterial and fungal infections was higher in the HLA-haploidentical group, which was consistent with a previous report^32^. Chang *et al*. suggested that the early delayed immune reconstitution after HLA-haploidentical HSCT probably led to an increased incidence of infection^33^. In the multivariate analysis, previous RBC transfusion was revealed to be an adverse factor for SAA patient survival after HSCT, which may be explained by decreased organ function due to iron deposition prior to HSCT. Although iron chelation therapy was administered to these patients before HSCT, the impact of RBC transfusion seemed to be irreversible. This is also the reason why upfront HLA-haploidentical HSCT is the preferred treatment. The GVHD prophylaxis regimens also seemed to have an impact on survival, but the number of patients in the PT-CY subgroup were too small to draw a persuasive conclusion.

In this study, all the SAA patients were given timely intravenous immunoglobulin (IVIG) as a basic infection prevention therapy. Only 23.6% of the patients in the HLA-haploidentical group and 14.7% of the patients in the HLA-identical group developed CMV infections; these rates were significantly lower than those in another SAA HLA-haploidentical HSCT study without the use of timely IVIG^34^. Although our systematic review and meta-analysis showed no advantage from IVIG for the prevention of CMV infection^35^, we still believe that IVIG plays an important role in the prevention of CMV infection among SAA patients after allogeneic HSCT. Indeed, that meta-analysis included only patients with hematologic malignancy, and SAA was not within the scope of the systematic review.

The baseline analysis before PSM showed that patients in the HLA-haploidentical HSCT group were significantly younger and that their donors were older than those in the HLA-identical HSCT group. Due to the one-child policy in China over the past 30 years, parent donors accounted for 77.4% (89/115) of the HLA-haploidentical group. The difference in the patient ages was either due to the one-child policy or the preference of the doctors and patients, as the previously prevailing opinion was that younger patients had better outcomes. Because the present study was not a randomized controlled trial, the doctors’ and patients’ choice of conditioning regimens was based on the knowledge that HLA-haploidentical transplantation had a higher graft rejection rate. Thus, more intensive conditioning regimens and sufficient immunosuppression were required. As a result, the patients in the HLA-haploidentical group were more likely to receive the Bu + CY conditioning regimen than the patients in the HLA-identical group. Similarly, FK506-based GVHD prophylaxis was more often used in the HLA-haploidentical group, as those patients were believed to be at greater risk of severe GVHD. Due to the retrospective nature of this study, we could not control for selection bias and exposure factors (e.g., the diverse conditioning regimens and GVHD prophylaxis regimens). Thus, we performed PSM to reduce selection bias and other potential influences. The results from the cohort after matching were similar to the results obtained before matching. However, there are still limitations in this study. Due to the relatively small sample sizes, subgroup analyses were not performed for the different conditioning regimens that were observed to affect OS or for the different age groups that showed an effect on GVHD and infection incidence. These results should therefore be validated by a well-designed, multicenter prospective study with a larger sample size.

In summary, this clinical study suggests that HLA-haploidentical HSCT achieves outcomes comparable to those of HLA-identical sibling HSCT for SAA patients younger than 40 years. HLA-haploidentical relative HSCT should be considered as a valid alternative option for patients younger than 40 years with SAA for whom no matched sibling donor is available.

## Methods

### Patients

From January 2006 to December 2015, 189 consecutive patients with SAA who underwent HLA-identical or HLA-haploidentical HSCT at seven transplant centers in western China were enrolled in this study. The SAA diagnosis was based on bone marrow cytomorphology, bone marrow biopsy, and karyotyping according to the Education Program of the American Society of Hematology^36^. All of the patients met the following criteria: (1) voluntary participation in HSCT, including the provision of written informed consent from the patients or their guardians, and (2) the absence of uncontrolled infections and severe liver, renal, lung and heart diseases. Patients with ferritin levels greater than 1000 ng/mL were treated with deferoxamine until the ferritin concentration was less than 1000 ng/mL before undergoing HSCT. This study was approved by the ethics committees of the Second Affiliated Hospital of Third Military Medical University and was conducted in accordance with the Declaration of Helsinki.

### Donors and graft sources

All patients described in this study received stem cells from their living relatives (siblings, parents or adult children). HLA class I (A, B and C) and HLA class II (DRB1 and DQB1) high-resolution typing was performed for donor selection. For the HLA-haploidentical group, 2 to 5 loci were mismatched. Peripheral blood stem cells (PBSCs) and bone marrow mononuclear cells (BMNCs) were mobilized and collected using standard protocols37. Stem cells for transplantation were collected at seven transplantation centers, including Department of Hematology, Xinqiao Hospital, Third Military Medical University; Department of Hematology, General Hospital of Kunming Military Region of PLA; Department of Hematology, Affiliated Hospital of Guizhou Medical University; Department of Hematology, Tangdu Hospital, Forth Military Medical University; Department of Hematology, General Hospital of Chengdu Military Region of PLA; Department of Hematology, First Yunnan Provincial People’s Hospital; and Department of Hematology, Affiliated Hospital of Kunming Medical College. No organs/tissues were procured from prisoners.

### Conditioning regimen

The patients underwent 3 types of conditioning regimens: (1) FAC: fludarabine (Flu) 30 mg/m^2^ daily i.v. on days −5 to −2, cyclophosphamide (CY) 45 mg/kg once daily i.v. on days −3 and −2, and antithymocyte globulin (ATG) 2.5 mg/kg once daily i.v. on days −5 to −2; (2) BU + CY + ATG: busulfan (BU) 0.8 mg/kg q6h i.v. on days −7 to −4, CY 60 mg/kg once daily on days −3 to −2, and ATG 2.5 mg/kg once daily i.v. on days −5 to −2; and (3) PT-CY: Flu 40 mg/kg once daily i.v. on days −5 to −2 and CY 1.8 g/m^2^ once daily on days −5 to −2 and +3 to +4.

### GVHD prophylaxis and treatment

**Figure 7:**
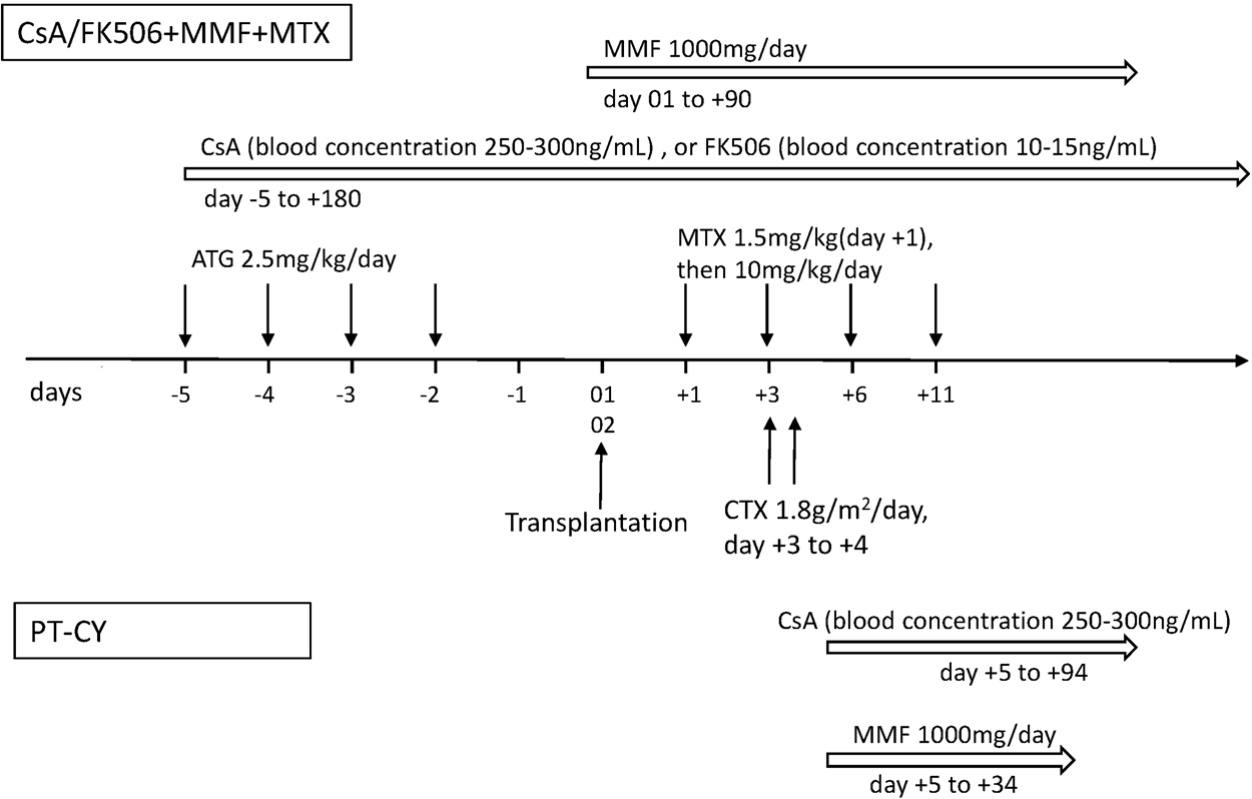
Graft versus host disease prophylaxis regimens.

All of the patients, except those in the PT-CY group, received cyclosporine A (CsA) or tacrolimus (FK506) plus mycophenolate mofetil (MMF) and short-term methotrexate (MTX) for acute GVHD (aGVHD) prophylaxis. HLA-haploidentical HSCT patients from the PT-CY group also received CsA plus MMF, whereas the HLA-identical HSCT patients used only CY (the detailed treatments are described in Fig. 7). aGVHD was defined according to the Fred Hutchinson Cancer Research Center criteria and was treated with 1–2 mg/kg of methylprednisolone per day. Second-line IST, such as CD25 monoclonal antibody (MoAb) (daclizumab; Roche, Basel, Switzerland) or MTX, was given for steroid-refractory aGVHD. Chronic GVDH (cGVHD) was diagnosed according to the 2014 NIH criteria^37^ and was treated with 1–2 mg/kg per day of prednisolone equivalents and a full-dose of CsA or FK506.

### Supportive care

All of the patients lived in a laminar air-flow room from day −8 to hematopoietic reconstitution and received prophylactic antibiotics when their absolute neutrophil count (ANC) was <0.5 × 10^9^ cells/L. Norfloxacin, trimethoprim sulfamethoxazole and ganciclovir were routinely administered according to a previously reported method^38^, and micafungin (50 mg/day), itraconazole (p.o. 200 mg/day) or voriconazole (p.o. 100 mg/day) was administered from day −1 to prevent invasive fungal infections (IFIs). Intravenous immunoglobulin (IVIG 0.4 g/kg once weekly before +100 days and 0.4 g/kg once monthly after +100 days) was given to increase passive immunity. CMV DNA was monitored weekly by PCR. CMV-positive patients were treated with either ganciclovir or foscarnet. Irradiated and filtered red blood cell and platelet transfusions were given to maintain a hemoglobin (Hb) level >80 g/L and a platelet count >20 × 10^9^/L. All of the patients received recombinant human granulocyte colony stimulating factor (G-CSF) from day 01 after hematopoietic stem cell infusion until hematopoietic reconstitution.

### Propensity score matching

Patient allocation in this study was based on the HLA-identical or HLA-haploidentical group assignments rather than by random assignment; therefore, the baseline levels of some clinical characteristics were imbalanced between the two groups (see Table 1). To reduce the influence of potential confounders, propensity score matching (PSM) was applied in this study^39,40^.

The propensity score that indicated the HLA status for each patient was calculated based on a multivariate logistic regression model. In this model, the dependent variable was a dichotomous variable [the HLA status (identical or haploidentical)], and the previously mentioned imbalanced variables between the two groups were used as covariates (patient and donor age, conditioning regimen, and GVHD prophylaxis). Patients in the HLA-identical group were matched to those in the HLA-haploidentical group using 1:1 nearest neighbor matching with a caliper width of 0.2.

### Statistical analysis

The primary endpoint of the study was overall survival (OS). The secondary endpoints included the incidences of GVHD and infection and the time to engraftment. The time at which PBSCs and BMNCs were infused was the starting point for comparing the outcomes between cohorts. The date of the last follow-up for all surviving patients was January 1, 2015. The median follow-up time was 20.35 months (range: 1–102 months).

Categorical data were summarized as counts and percentages, whereas continuous data were expressed as medians and ranges. Comparisons of the baseline and transplant-related characteristics between the 2 groups were performed before and after PSM using the χ2 test for categorical variables and the non-parametric Mann-Whitney test for continuous variables. Survival functions and the cumulative incidence of aGVHD or cGVHD were estimated by the Kaplan-Meier method and compared using log-rank tests. Multivariate Cox stepwise regression was used to determine the relationship between survival and risk factors; P < 0.05 was considered to indicate statistical significance.

A statistical software package (IBM SPSS Statistics 22, IBM Corp., Armonk, NY, USA) was used for the analyses.

## Electronic supplementary material

Supplementary Information
