# Supplementary material for: Optimal donor for severe aplastic anemia patient requiring allogeneic hematopoietic stem cell transplantation: A large-sample study from China
Source: Sci Rep. 2018 Feb 6;8:2479. doi: 10.1038/s41598-018-20853-9 (PMC5802708; doi:10.1038/s41598-018-20853-9)
Supplement: Supplementary file 1 — Supplementary Information [file 41598_2018_20853_MOESM1_ESM.doc]

# Optimal donor for severe aplastic anemia patient requiring allogeneic hematopoietic stem cell transplantation: A large-sample study from China

Yunjing Zeng^1^, Sanbin Wang^2*^, Jishi Wang^3^, Li Liu^4^, Yi Su^5^, Zhixiang Lu^6^, Xuemei Zhang^7^, Yanqi Zhang^8^, Jiang Fan Zhong^1^,Lihui Peng^2^, Qiang Liu^4^, Yinghao Lu^3^, Lei Gao^1#^, Xi Zhang^1#^

^1^Department of Hematology, Xinqiao Hospital, Third Military Medical University, Chongqing, China

^2^Department of Hematology, General Hospital of Kunming Military Region of PLA, Kunming, China

^3^Department of Hematology, Affiliated Hospital of Guizhou Medical University, Guiyang, China

^4^Department of Hematology, Tangdu Hospital, Forth Military Medical University, Xi’an, China

^5^Department of Hematology, General Hospital of Chengdu Military Region of PLA, Chengdu, Sichuan, China

^6^Department of Hematology, First Yunnan Provincial People’s Hospital, Kunming, China

^7^Department of Hematology, Affiliated Hospital of Kunming Medical College, Kunming, China

^8^Department of Health Statistics, College of Military Preventive Medicine, Third Military Medical University, Chongqing, China

^#^Corresponding author: Xi Zhang and Lei Gao

Department of Hematology

Xinqiao Hospital

Third Military Medical University

Chongqing, China 400037

Tel.: 86-23-68755609 Fax: 86-23-68763198

E-mail: [zhangxxi@sina.com](mailto:zhangxxi@sina.com); gaolei7765@163.com

^*^This author is the co-first author.

Table S1. Univariate Cox regression of risk factors on survival in transplantation patients with severe aplastic anemia

| **characteristics** | **Before PSM** | | |  | **After PSM** | | |
| --- | --- | --- | --- | --- | --- | --- | --- |
|  | *P* value | HR | 95.0% CI of HR |  | *P* value | HR | 95.0% CI for HR |
| Identical group | 0.211 | 0.640 | 0.318-1.287 |  | 0.162 | 0.587 | 0.278-1.238 |
| Patients age | 0.233 | 1.331 | 0.832-2.128 |  | 0.133 | 1.500 | 0.884-2.548 |
| Female patient | 0.991 | 0.996 | 0.505-1.967 |  | 0.985 | 0.993 | 0.474-2.079 |
| Interval from diagnosis to HSCT | 0.133 | 1.006 | 0.998-1.013 |  | 0.047 | 1.007 | 1.000-1.014 |
| Donor age | 0.120 | 1.026 | 0.993-1.059 |  | 0.196 | 1.022 | 0.989-1.056 |
| Female donor | 0.037 | 0.446 | 0.228-0.954 |  | 0.112 | 0.528 | 0.240-1.160 |
| ABO blood type | 0.555 | 1.093 | 0.813-1.470 |  | 0.793 | 1.048 | 0.736-1.494 |
| PBSC infused cell dose | 0.857 | 1.008 | 0.929-1.093 |  | 0.835 | 1.011 | 0.916-1.115 |
| BMNC infused cell dose | 0.682 | 0.969 | 0.834-1.127 |  | 0.725 | 1.029 | 0.878-1.205 |
| CD34^+^cells infused cell dose | 0.637 | 1.021 | 0.936-1.114 |  | 0.584 | 1.027 | 0.934-1.129 |
| [Conditioning regimen](http://cn.bing.com/dict/clientsearch?mkt=zh-CN&setLang=zh&form=BDVEHC&ClientVer=BDDTV3.5.0.4311&q=预处理方案) | 0.333 | 0.741 | 0.404-1.359 |  | 0.229 | 0.631 | 0.298-1.335 |
| GVHD prophylaxis | 0.020 | 1.616 | 1.078-2.424 |  | 0.023 | 1.713 | 1.076-2.726 |
| Previous RBC transfusion | 0.021 | 1.017 | 1.003-1.032 |  | 0.003 | 1.024 | 1.008-1.041 |
| Previous PLT transfusion | 0.234 | 1.006 | 0.996-1.015 |  | 0.639 | 1.003 | 0.991-1.014 |
| Previous ANC | 0.484 | 0.509 | 0.077-3.382 |  | 0.408 | 0.415 | 0.052-3.325 |
| Previous HGB | 0.767 | 0.996 | 0.972-1.021 |  | 0.510 | 0.991 | 0.964-1.018 |
| Previous PLT | 0.123 | 1.047 | 0.988-1.110 |  | 0.287 | 1.035 | 0.972-1.102 |
| Previous RET | 0.559 | 1.016 | 0.964-1.070 |  | 0.614 | 1.015 | 0.958-1.075 |
| Previous IST | 0.126 | 2.094 | 0.813-5.392 |  | 0.021 | 3.136 | 1.188-8.277 |

HSCT: hematopoietic stem cell transplantation; PBSC, peripheral blood stem cell; BMSC, bone marrow stem cell; GVHD: graft versus host disease; RBC, red blood cells; PLT, platelets; HGB, hemoglobin; CBCs: complete blood counts; ANC: absolute neutrophil count; RET: reticulocyte count; IST: immunosuppressive therapy.

Table S2. Multivariate Cox regression of risk factors on survival in transplantation patients with severe aplastic anemia

| **characteristics** | **Before PSM** | | |  | **After PSM** | | |
| --- | --- | --- | --- | --- | --- | --- | --- |
|  | *P* value | HR | 95.0% CI of HR |  | *P* value | HR | 95.0% CI for HR |
| Identical group | 0.505 | 0.509 | 0.070-3.708 |  | 0.213 | 0.191 | 0.014-2.585 |
| Patients age | 0.975 | 1.017 | 0.353-2.933 |  | 0.231 | 0.345 | 0.061-1.967 |
| Female patient | 0.664 | 1.421 | 0.292-6.920 |  | 0.984 | 0.976 | 0.090-10.637 |
| Interval from diagnosis to HSCT | 0.479 | 0.985 | 0.946-1.026 |  | 0.961 | 1.001 | 0.967-1.036 |
| Donor age | 0.205 | 1.045 | 0.976-1.119 |  | 0.370 | 1.052 | 0.942-1.174 |
| Female donor | 0.221 | 0.352 | 0.066-1.871 |  | 0.636 | 0.531 | 0.039-7.282 |
| ABO blood type | 0.631 | 0.826 | 0.378-1.803 |  | 0.688 | 0.766 | 0.209-2.807 |
| PBSC infused cell dose | 0.477 | 0.899 | 0.671-1.205 |  | 0.989 | 0.997 | 0.672-1.481 |
| BMNC infused cell dose | 0.220 | 0.814 | 0.587-1.131 |  | 0.211 | 0.640 | 0.319-1.287 |
| CD34^+^cells infused cell dose | 0.889 | 0.988 | 0.839-1.164 |  | 0.722 | 0.930 | 0.624-1.386 |
| [Conditioning regimen](http://cn.bing.com/dict/clientsearch?mkt=zh-CN&setLang=zh&form=BDVEHC&ClientVer=BDDTV3.5.0.4311&q=预处理方案) | 0.606 | 1.652 | 0.246-11.096 |  | 0.647 | 1.700 | 0.175-16.497 |
| GVHD prophylaxis | 0.001 | 36.652 | 4.465-300.885 |  | 0.026 | 124.131 | 1.796-8580.394 |
| Previous RBC transfusion | 0.997 | 1.000 | 0.949-1.054 |  | 0.345 | 0.937 | 0.819-1.072 |
| Previous PLT transfusion | 0.123 | 1.018 | 0.995-1.042 |  | 0.338 | 1.029 | 0.971-1.090 |
| Previous ANC | 0.037 | 0.008 | 0.000-0.741 |  | 0.314 | 0.037 | 0.000-22.860 |
| Previous HGB | 0.759 | 0.992 | 0.945-1.042 |  | 0.157 | 0.936 | 0.854-1.026 |
| Previous PLT | 0.098 | 1.126 | 0.978-1.297 |  | 0.287 | 1.109 | 0.917-1.342 |
| Previous RET | 0.606 | 1.033 | 0.913-1.170 |  | 0.134 | 1.128 | 0.964-1.319 |
| Previous IST | 0.333 | 4.364 | 0.221-86.259 |  | 0.691 | 3.010 | 0.013-685.022 |

HSCT: hematopoietic stem cell transplantation; PBSC, peripheral blood stem cell; BMSC, bone marrow stem cell; GVHD: graft versus host disease; RBC, red blood cells; PLT, platelets; HGB, hemoglobin; CBCs: complete blood counts; ANC: absolute neutrophil count; RET: reticulocyte count; IST: immunosuppressive therapy.
